# Accurate placement of substrate RNA by Gar1 in H/ACA RNA-guided pseudouridylation

**DOI:** 10.1093/nar/gkv757

**Published:** 2015-07-22

**Authors:** Peng Wang, Lijiang Yang, Yi Qin Gao, Xin Sheng Zhao

**Affiliations:** College of Chemistry and Molecular Engineering, Beijing National Laboratory for Molecular Sciences, State Key Laboratory for Structural Chemistry of Unstable and Stable Species, and Biodynamic Optical Imaging Center (BIOPIC), Peking University, Beijing 100871, China

## Abstract

H/ACA RNA-guided ribonucleoprotein particle (RNP), the most complicated RNA pseudouridylase so far known, uses H/ACA guide RNA for substrate capture and four proteins (Cbf5, Nop10, L7Ae and Gar1) for pseudouridylation. Although it was shown that Gar1 not only facilitates the product release, but also enhances the catalytic activity, the chemical role that Gar1 plays in this complicated machinery is largely unknown. Kinetics measurement on *Pyrococcus furiosus* RNPs at different temperatures making use of fluorescence anisotropy showed that Gar1 reduces the catalytic barrier through affecting the activation entropy instead of enthalpy. Site-directed mutagenesis combined with molecular dynamics simulations demonstrated that V149 in the thumb loop of Cbf5 is critical in placing the target uridine to the right position toward catalytic D85 of Cbf5. The enzyme elegantly aligns the position of uridine in the catalytic site with the help of Gar1. In addition, conversion of uridine to pseudouridine results in a rigid syn configuration of the target nucleotide in the active site and causes Gar1 to pull out the thumb. Both factors guarantee the efficient release of the product.

## INTRODUCTION

The modification of uridine (U) to pseudouridine (Ψ) is the most abundant RNA modification in transfer RNA (tRNA), ribosomal RNA (rRNA), and small nuclear RNA (snRNA) (1). Ψ can stabilize RNA structure (2–5), maintain the balance between the flexibility and stability of tRNA anticodon stem loop (6), modulate ribosome synthesis (7–9), and even convert nonsense codons into sense codons (10,11). Pseudouridine synthases (ΨSs) catalyze the site-specific conversion of U to Ψ in RNA (12). The modification involves the cleavage of the N_1_-glycosidic bond in the target U, the rotation of the base, and the formation of a C_5_-glycosidic bond. ΨSs are classified into six families named after the representative members: TruA, TruB, RluA, RsuA, TruD and Pus10. Structure studies have shown that all ΨSs share a catalytic domain with a similar fold and a conserved active-site cleft and suggest a common catalytic mechanism. All ΨSs contain a strictly conserved aspartic acid (Asp85 in *Pyrococcus furiosus* Cbf5) in the active site. The key Asp is essential for catalysis in all ΨSs, but the exact catalytic mechanism remains unclear. It may act as a nucleophile to attack C_6_ of the uracil ring to form a Michael adduct or to attack on the ribose ring to form either an acylal intermediate or a glycal intermediate (13–16).

ΨSs recognize the substrate using two different mechanisms. All ΨSs, except for the H/ACA RNA-guided ribonucleoprotein particle (RNP), are single polypeptide stand-alone enzymes and recognize the substrate through the protein-RNA interface. In contrast, RNP is composed of a distinct H/ACA guide RNA and four conserved partner proteins Cbf5, Nop10, L7Ae and Gar1 (17–20). By implementing guide RNA, RNP recognizes the substrate through RNA-RNA interaction. Each H/ACA RNA has a hairpin-hinge-hairpin-tail structure with a large internal loop (1). The loop can form two ∼6-bp duplexes with the substrate and creates a pseudouridylation pocket in which the target U is unpaired and thus flanked to be modified (21). In eukaryotes, H/ACA RNAs are composed of two hairpins, and each hairpin constitutes the basic structural and functional unit in vitro (22). Cbf5 is the catalytic subunit and shares significant sequence similarity with TruB, the stand-alone bacterial tRNA Ψ55 synthase (23). It is believed that Cbf5 shares the same catalytic mechanism as TruB and they differ only in the substrate recognition mechanism. Cbf5 may also act as a stand-alone pseudouridine synthase on tRNA substrates (24–26).

An assembly of all components of RNP is required for an optimal pseudouridylation activity and RNP stability (27–31). L7Ae can remodel the conformation of guide RNA and then place the target U into the active site of Cbf5 through the concurrent interaction of L7Ae with the kink-turn motif of the guide RNA and with the composite surface formed by Nop10 and Cbf5 (32). Nop10 and Gar1 are distant from the catalytic site of Cbf5, which precludes their direct catalytic roles. The crystal structure of the Cbf5-Nop10 complex suggests that Nop10 can buttress the active site of Cbf5 and organize the binding of guide RNA-substrate RNA complex by extending the active site cleft (33). Gar1 was proposed to facilitate product release by pulling the thumb loop off from the product (34). However, it is difficult to explain the catalytic promotion of Gar1 in single turnover reactions that do not require product release (34–37). Furthermore, Gar1 was also shown to remodel substrate RNA that is misdocked with partially assembled RNPs (38).

Because the crystal structure of the complex of RNP with neither natural reactant nor product can be obtained, the complex structure was studied on the complex of RNP with 5-fluoro-6′-hydroxyl pseudouridine (5FhoΨ) which was converted from 5-fluorouridine by the enzyme. 5FhoΨ associates with the pseudouridylation pocket and adopts a U shape that is aligned vertically relative to the protein surface (34,39). The thumb loop adopts an open conformation in the absence of substrate RNA with its tip region disordered and its N-terminal root region docked at Gar1 (40). When RNP binds with 5FhoΨ, the thumb loop switches to a closed conformation and interacts extensively with 5FhoΨ (Supplementary Figure S1A), which is regarded to mimic most closely the transition state (34,39). The interactions between the thumb loop and Gar1 are mediated mainly through the N-terminal segment of the former (residues 140–145) (34). The rest of the thumb loop binds 5FhoΨ, and the tip region of the thumb loop fits neatly into a complementary space between the 5′ and 3′ arms of the substrate RNA in the closed state (34). Notably, the interaction of the thumb loop with the floor of the Cbf5 D2 subdomain can drive the initial flipping of the thumb loop when the substrate RNA is not fully in place (41). Deletion of the thumb loop caused a complete loss of the enzyme activity of RNP (34). However, it is unclear to what extent the RNP crystal structure obtained with the substrate analogues reflects the true conformation and interaction between RNP and its natural substrates.

Previously, we used fluorescence correlation spectroscopy (FCS) measurement to interrogate the kinetics of U to Ψ conversion (37). We found that the thumb loop is required for the substrate RNA–enzyme complex to form its most stable state but has a minor effect on the release of product RNA. RNP can distinguish between U and Ψ, so that the release of the modified product is faster than that of the reactant despite of their little structural difference. Gar1 accelerates greatly the product but slightly the reactant release. Most importantly, Gar1 enhances significantly the reaction rate of the modification step. Since Gar1 has no direct contact with the substrate RNA or the catalytic center, it was speculated that Gar1 helps the thumb loop to maintain an optimal configuration with respect to the substrate RNA, which reduces the activation barrier of the reaction (37). However, the molecular details are unknown.

In this study, we aim at understanding the chemistry through which Gar1 enhances the discrimination between U and Ψ and accelerates the modification reaction. We studied the temperature dependence of the rates of association and dissociation of the reactant and product, as well as the catalytic rate for the modification of U to Ψ, using fluorescence anisotropy (FA) measurement. This technique allowed us to record the kinetic data with a bin time (0.1 s) smaller than the FCS measurement (30 s), so that we were able to monitor the processes closer than the FCS technique could (37). Combining site-directed mutagenesis experiments with molecular dynamics (MD) simulations, we were able to understand the interesting chemistry that enables RNP to maintain high efficiency for pseudouridylation while implementing the RNA-RNA recognition to achieve high flexibility.

## MATERIALS AND METHODS

### Protein mutagenesis, expression, purification and assembly of RNP

The full *Pyrococcus furiosus* RNP complex (WT-RNP) was assembled by mixing the Cbf5-Nop10 subcomplex, Gar1, guide RNA, and L7Ae in a 1:1:1:2 molar ratio in 50 mM phosphate buffer (pH 7.6, 1 M NaCl) at 37°C for 30 min (40). The ΔGar1 complex (ΔGar1-RNP) was assembled without Gar1. All mutants of Cbf5 were generated by site-directed mutagenesis with Fast Mutagenesis System Kit (TransGen. Biotech) according to the manufacturer's protocol. Each protein was individually expressed in *E. coli* Rosseta (DE3) cells grown in LB medium, but Cbf5 and Nop10 were co-purified as a dimer, as previously described (34,37,40).

### Substrate and product RNA

The cognate substrate RNA with uridine at the target site (Sub-U) was synthesized with a fluorescent dye DY547 attached to the 3′-end and the corresponding product RNA (Sub-Ψ) was prepared by enzymatic modification of Sub-U as previously described (37).

### Fluorescence anisotropy

FA measurements were performed on a home-built dual-channel confocal fluorescence microscope based on a TE2000 microscope (Nikon) equipped with a 532 nm solid-state laser (MLL-III-532, CNI) (42). The laser beam was vertically polarized by a polarizer and then focused inside the sample solution, through an oil immersion objective (NA 1.4, 100×, Nikon), 10 μm above the glass surface. The laser power was 300 μW. The fluorescence was separated from the excitation light by a dichroic mirror (Z532, Chroma). After being focused through a 30 μm pinhole, the fluorescence was separated into p-polarized and s-polarized components with a polarized beam splitter (PBS) (Daheng, China). Each component was detected by a photon-counting avalanche photodiode (APD) (SPCM-AQRH-14, Perkin-Elmer Optoelectronics) after passing through a filter (Semrock FF01-593/40). Fluorescence intensities were recorded with a photon counters card (PMS-400A, Becker & Hickl) in 100 ms bin time. The raw data was converted to *r*(*t*) with 1 s bin time using the following equation:

(1) \begin{equation*} r(t) = \frac{{I_{\rm p} (t) - I_{\rm s} (t)}}{{I_{\rm p} (t) + 2I_{\rm s} (t)}} \end{equation*}

where *I*_p_(*t*) and *I*_s_(*t*) are the fluorescence intensities of p-polarized and s-polarized components, respectively, in each 1 s bin.

### Kinetic association and dissociation experiments

**Figure 1.**
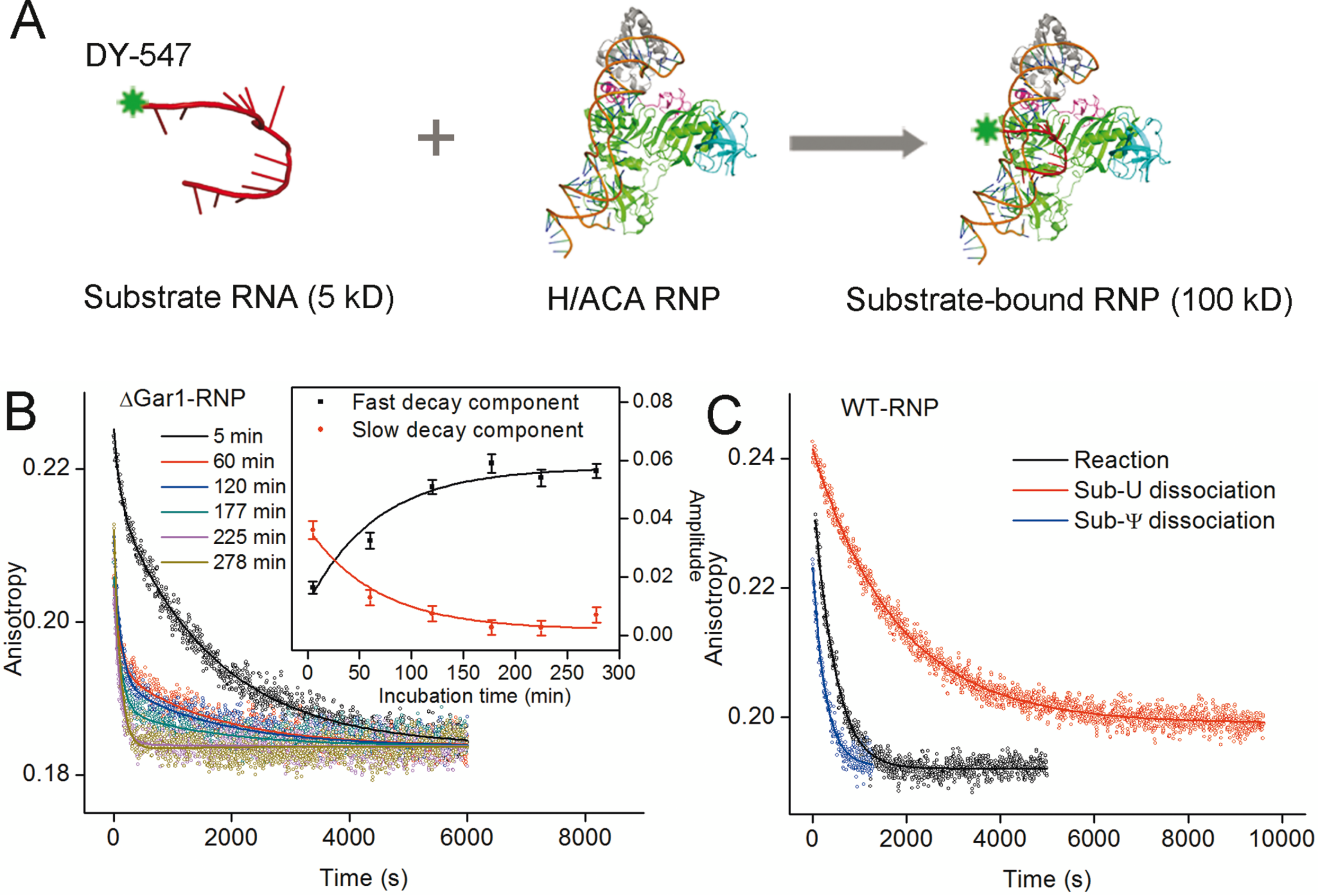
Catalytic modification rates measurement using FA. (**A**) Schematics of substrate RNA and RNP binding process. The large difference in molecular weights leads to a large difference in FA. The fluorescent dye DY547 is attached to the 3′-end of substrate RNA. (**B**) Dissociation curves after 4000-fold dilution of the Sub-U/ΔGar1-RNP reactive complexes which had been incubated for different times at 27°C. All curves were globally fitted to a double-exponential function. The two amplitudes were plotted against the incubation time and globally fitted to a single-exponential function to determine the catalytic modification rate (inset). (**C**) Dissociation curves of the Sub-U/WT-RNP reactive complex (black), Sub-U/D85A-RNP complex (red) and Sub-Ψ/WT-RNP complex (blue) at 22°C. The curves of Sub-U/D85A-RNP and Sub-Ψ/WT-RNP complexes were fitted to a single-exponential function respectively to derive dissociation rates of Sub-U and Sub-Ψ. Catalytic modification rate was determined by fitting the curve of Sub-U/WT-RNP complex to a double-exponential function with the dissociation rates of Sub-U and Sub-Ψ fixed.

All experiments were performed at desired temperatures maintained by a temperature controller (PE100-NISystem, Linkam). The nonreactive D85A-RNPs were used for association and dissociation experiments of Sub-U, because D85A-RNPs assembled using the D85A-Cbf5 mutants are catalytically inactive but remain almost identical to WT-RNP in terms of substrate association and dissociation (37). The free and RNP bound substrates show well separated FA due to their large difference in molecular weight (5 kD versus 100 kD, Figure 1). Detailed experimental procedures were previously described (37). Briefly, kinetic association experiments were performed by mixing equal volumes of RNP and DY547 labeled substrate (10 nM final concentration) and real-time monitoring the fluorescence signal. The association experiments were conducted with four different concentrations of enzyme (0.10–0.75 μM). The converted FA curves by Equation (1) were fitted to a single exponential function to derive the apparent association rates *k*_obs_. The *k*_obs_ values at different enzyme concentrations were fitted to the linear equation:

(2) \begin{equation*} k_{{\rm obs}} = k_{{\rm on}} [E] + k_{{\rm off}} \end{equation*}

where [*E*] is the concentration of RNP, *k*_on_ and *k*_off_ are the association and dissociation rates, respectively.

For the kinetic dissociation experiments, substrate RNA (2 μM) and RNP (6 μM) were incubated for 60 min. The mixture was then rapidly diluted with buffer (50 mM phosphate buffer, pH 7.6, 1 M NaCl) to a final substrate concentration of 0.5 nM. The fluorescence signal was continuously recorded until a plateau was reached. Dissociation rates *k*_off_ were derived by fitting the FA curves to a single exponential function.

### The measurement of catalytic rates

The FA measurement in current work followed the same principle as the previously described FCS method (37). The only difference is that the time resolution was improved by switching the method from FCS to FA. The pseudouridine formation catalyzed by RNP can be represented by


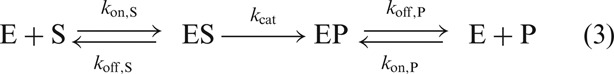


where S is the substrate (Sub-U), P is the modified product (Sub-Ψ), E is the enzyme, ES and EP are the most stable complexes prior to dissociation.

WT-RNP and ΔGar1-RNP are both active toward Sub-U. The extent of conversion of Sub-U to Sub-Ψ by RNP varies with the incubation time, and a longer incubation time led to the production of more Sub-Ψ. Therefore, The dissociation curve of substrate-enzyme complex monitors three concurrent processes: dissociation of unmodified substrate, dissociation of modified product, and modification, which can be fitted to a double-exponential function (37):

(4) \begin{equation*} r(t) = A_1 e^{ - k_1 t} + A_2 e^{ - k_2 t} \end{equation*}

where the fast decay rate *k*_1_ corresponds to the dissociation rate of Sub-Ψ (*k*_off,P_), the slow decay rate *k*_2_ is the sum of the modification rate (*k*_cat_) and the dissociation rate of Sub-U (*k*_off,S_), and *A*_1_ and *A*_2_ are the amplitudes. Because RNP/Sub-Ψ dissociates faster than RNP/Sub-U (37), the longer the incubation time, the sharper the decay curve is (Figure 1B).

For the measurement of *k*_cat_ of ΔGar1-RNP, Sub-U (2 μM) was incubated with RNP (6 μM) for different times prior to dilution. The mixture was then rapidly diluted with buffer (50 mM phosphate buffer, pH 7.6, 1 M NaCl) to a final substrate concentration of 0.5 nM. The fluorescence signal was continuously recorded until a plateau was reached. All dissociation curves with different incubation times were globally fitted to Equation (4) (Figure 1B). *k*_1_ and *k*_2_ were kept the same while *A*_1_ and *A*_2_ were variable. *k*_cat_ was determined by globally fitting *A*_1_ and *A*_2_ to a single-exponential function of the incubation time (Figure 1B, inset) (37). However, the modification rate of WT-RNP ((1.86 ± 0.10) × 10^−3^ s^−1^ at 22°C) is much faster than that of ΔGar1-RNP ((0.17 ± 0.08) × 10^−3^ s^−1^ at 22°C), and different time incubation is infeasible. For the determination of the WT-RNP modification rate, we recorded the dissociation curves of reactive WT-RNP enzyme-substrate complex after a short incubation time of 0.5 min and fit them using Equation (4). Parameters *k*_off,P_ and *k*_off,S_ determined from independent dissociation experiments were put in and fixed in the fitting process to derived *k*_cat_ (37) (Figure 1C).

At the enzyme concentration (6 μM) used in the measurement of the catalytic rate, the association processes were much faster than the modification processes. Therefore, the influence of binding to the measurement of the catalytic rate is negligible. For instance, at 15°C, the association half time of WT-RNP/Sub-U complexes was 29 s, which was about the same as our experimental dead time of data acquisition due to the hand mixing. Meanwhile the catalytic half time was 45 min. At elevated temperatures, the situation that association processes were much faster than the modification processes held throughout, with the association half time progressively becoming shorter. For ΔGar1-RNP and all the mutations, the situation is even better than the case of WT-RNP due to their reduced catalytic activities but similar binding properties to that of WT-RNP. The reliability and accuracy of the FA measurement was confirmed by comparing the catalytic rates obtained among current FA and previous FCS and more traditional thin layer chromatography (37). The modification rate of WT-RNP at 27°C measured by FA ((5.4 ± 0.5) × 10^−3^ s^−1^) was comparable with that determined by the single turnover activity assay ((3.9 ± 0.6) × 10^−3^ s^−1^), and the measured modification rate of ΔGar1-RNP by FA ((0.24 ± 0.08) × 10^−3^ s^−1^) was in agreement with that measured by FCS ((0.19 ± 0.05) × 10^−3^ s^−1^) (37).

### Molecular dynamics simulations

All MD simulations were performed using AMBER 12 package (AMBER 12, 2012). The proteins and RNA were modeled with AMBER FF10 all-atom force field and TIP3P water potential was used to model solvation. In these simulations, the SHAKE (43) algorithm with a relative geometric tolerance of 10^−5^ was used to constrain all chemical bonds. Thus, all dynamics utilized a 2 fs time step. Long-range electrostatics was treated by the particle-mesh Ewald (PME) (44) method with default settings and a 10 Å direct space nonbonded cutoff was used in all simulations. The initial structures of MD simulations are all based on the crystal structure of the close form RNP complex (PDBID: 3HAY). In each trajectory, the initial structure of RNP complex was first subjected to 2500 steps of minimization, and then the temperature of the system was established by velocity rearrangement from a Maxwell-Boltzmann distribution at 300 K. After these preparing steps the system was maintained at 300 K using the Langevin dynamics with a coupling constant of 2 ps^−1^. The snapshots used in MM/PBSA (45) calculations were taken from MD simulations trajectories of WT-RNP and ΔGar1-RNP complex with Sub-U and Sub-Ψ. The other options used in Poisson–Boltzmann calculations were set to the default values.

## RESULTS

### Gar1 reduces the activation free energy barrier of modification entropically instead of enthalpically

To reveal how Gar1 promotes the catalytic reaction, we employed FA to measure the catalytic rates of RNP at different temperatures by monitoring the change of the apparent molecular weight of the system as the FCS method does (37). The kinetics of modification by ΔGar1-RNP and WT-RNP were examined by following the anisotropy change in real time after dilution of the reactive ΔGar1-RNP/Sub-U and WT-RNP/Sub-U complexes, respectively (Figure 1B and C). Although release of both the reactant and product involves more than one step, our control experiments of D85A-RNP/Sub-U and RNP/Sub-Ψ previously and in this article showed that their dissociation curves can be fitted by single exponential function respectively, and it was also true in the absence of Gar1 (37). Therefore, the modification rate (*k*_cat_) can be obtained using the method described in the experimental section. Due to the high reactivity of WT-RNP, the modification processes were too fast to be monitored at temperatures over 22°C, so the modification rates were measured at the temperature range of 15–22°C. On the other hand, we found that the appropriate temperature range to measure the modification rates of ΔGar1-RNP was 22–37°C because of the lower reactivity of ΔGar1-RNP.

The data were fitted to the Arrhenius equation

**Figure 2.**
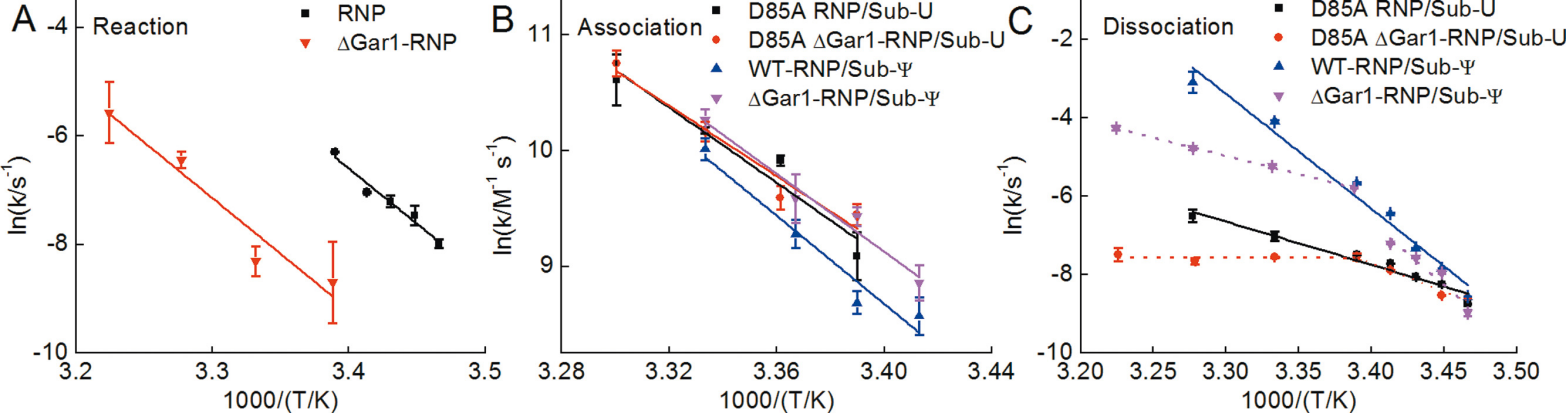
Apparent activation energies determined for reaction (**A**), association (**B**) and dissociation (**C**). Temperature dependent rates were fitted by the Arrhenius equation to derive the apparent activation energy. The transition temperature for the activation energies in the dissociation of D85A ΔGar1-RNP/Sub-U and ΔGar1-RNP/Sub-Ψ complex is about 22°C.

(5) \begin{equation*} k_{{\rm cat}} = Ae^{ - E_{\rm a} /RT} \end{equation*}

where *A* is the pre-exponential factor, *E*_a_ is the apparent activation energy, *R* is the gas constant, and *T* is the absolute temperature. The results are presented in Figure 2A and Supplementary Table S1. Intriguingly, the apparent activation energies of WT-RNP and ΔGar1-RNP for the modification are the same (40.4 ± 4.5 and 40.7 ± 7.1 kcal mol^−1^, respectively), and Gar1 only affects the pre-exponential factors. According to the transition state theory,

(6) \begin{equation*} k_{{\rm cat}} = \frac{{k_{\rm B} T}}{h}e^{ - \Delta G_{\rm a} /RT} = \frac{{k_{\rm B} T}}{h}e^{\Delta S_{\rm a} /R} e^{ - \Delta H_{\rm a} /RT} \end{equation*}

where Δ*G*_a_ is the activation free energy, Δ*S*_a_ is the activation entropy, Δ*H*_a_ is the activation enthalpy, *k*_B_ is the Boltzmann constant, and *h* is the Planck constant. *E*_a_ and *A* are related to Δ*H*_a_ and Δ*S*_a_ respectively by

(7) \begin{equation*} \begin{array}{*{20}c} {E_{\rm a} = \Delta H_{\rm a} + RT}  \\ {A = \frac{{k_{\rm B} T}}{h}\frac{e}{{c^\circ }}e^{\frac{{\Delta S_{\rm a} }}{R}} }  \\ \end{array} \end{equation*}

where *c*° is the unit concentration. The result, therefore, shows that Gar1 promotes the catalytic reaction entropically instead of enthalpically.

The temperature dependence of the association rate constant is similar for Sub-U and Sub-Ψ and is independent of Gar1 (Figure 2B). These results are consistent with the scenario in which the association is mainly controlled by the base pairing between guide RNA and Sub-U or Sub-Ψ and insensitive to other factors during the substrate loading (37). The measured dissociation rate constant is quite different for WT-RNP and ΔGar1-RNP (Figure 2C). In the case of ΔGar1-RNP, the temperature dependences are non-Arrhenius for both Sub-U and Sub-Ψ. At the temperature range of 15–22°C, there are appreciable activation barriers (Figure 2C, dotted line). As the temperature increases, the dissociation exhibits a lower activation barrier. The switching of the activation energy suggests that the reaction is kinetically controlled <22°C but diffusionally controlled >22°C.

### V149 is critical for the catalytic modification

The DEL7 mutation, of which 10 amino acid residues (143–152) in the tip region of the thumb loop were replaced by a sequence of glycine-proline-glycine, has been shown to completely abolish the activity (34,37). Here, we applied site-directed single point mutagenesis to the thumb loop to assess its effect on the enzymatic activity. We chose six residues (R146, S147, A148, V149, K150 and R151) in the tip region of the thumb loop and replaced one of them with glycine each time to generate single point mutants. These mutant proteins were still capable of assembling into full and ΔGar1-RNP complexes, as confirmed by native PAGE assay. We assessed their loading, unloading and catalytic activities using the FA kinetics measurements as described above. The experiments were performed at 27°C and 37°C for ΔGar1-RNPs in association and dissociation respectively, and 22°C for full RNPs. The association and dissociation rates are shown in Supplementary Table S2. Several general trends emerge from Supplementary Table S2. The single point mutation resulted in slight changes in the association rates of Sub-U and Sub-Ψ for both full- and ΔGar1-RNPs, but the changes were within an order of magnitude. For instance, compared with the association rate of WT-RNP/Sub-U ((8.8 ± 1.6) × 10^−3^ M^−1^ s^−1^), the largest difference occurred in V149L RNP/Sub-U ((3.9 ± 0.2) × 10^−3^ M^−1^ s^−1^), but the difference is <3-fold. A similar phenomenon was observed for the dissociation rates. These facts indicate that single point mutation has a minor effect on substrate loading and unloading.

**Figure 3.**
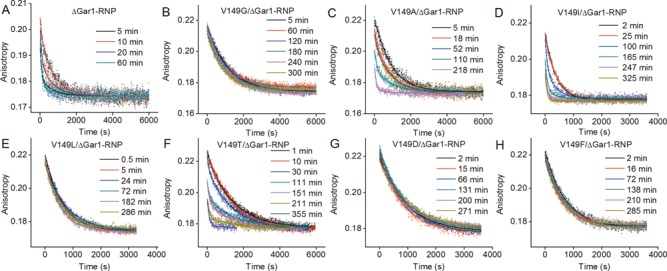
Dissociation curves of complexes of Sub-U with different ΔGar1-RNP mutants. (**A**) ΔGar1-RNP, (**B**) V149G/ΔGar1-RNP, (**C**) V149A/ΔGar1-RNP, (**D**) V149I/ΔGar1-RNP, (**E**) V149L/ΔGar1-RNP, (**F**) V149T/ΔGar1-RNP, (**G**) V149D/ΔGar1-RNP, (**H**) V149F/ΔGar1-RNP. The dissociation data for complexes of Sub-U with ΔGar1-RNP, V149A/ΔGar1-RNP, V149I/ΔGar1-RNP and V149T/ΔGar1-RNP were globally fitted to a double-exponential function to derive catalytic modification rates. The other curves were fitted to a single exponential function.

When one examines the catalytic reaction, the situation became noticeably different. Because the dissociation rates of Sub-U and Sub-Ψ were similar for the full-RNP mutants (Supplementary Table S2), we were unable to apply our methods to measure their catalytic rates. On the other hand, our strategy still worked well on the ΔGar1-RNP mutants. Measurements showed that the ΔGar1-RNP complexes assembled by Cbf5 mutants R146G, S147G, A148G, K150G and R151G still catalyze the conversion of U to Ψ although with reduced activities relative to ΔGar1-RNP (Supplementary Table S3). However, the dissociation curves of V149G ΔGar1-RNP/Sub-U complexes at all incubated times were the same within the experimental error (Figure 3B). Single exponential fitting of these dissociation curves of V149G ΔGar1-RNP/Sub-U complexes offered a mean dissociation rate of (1.13 ± 0.08) × 10^−3^ s^−1^ which is comparable to the Sub-U dissociation rate ((1.80 ± 0.05) × 10^−3^ s^−1^) measured using D85A/V149G ΔGar1-RNP, but much smaller than the Sub-Ψ dissociation rate ((8.6 ± 0.4) × 10^−3^ s^−1^, Supplementary Table S2). These results indicate that the V149G mutation causes the complete loss of the catalytic activity for ΔGar1-RNP within the longest incubation time of 5 h. V149 is strongly preserved in the tip of the thumb loop and only interacts with the substrate RNA through van der Waals contacts (34) (also see Supplementary Figure S1B). It is intriguing why such an inert residue is so critical. Next, we replaced V149 with alanine which has a larger side chain than glycine (but still smaller than valine), or leucine which has a larger side chain than valine. V149A ΔGar1-RNP partially recovers the catalytic activity while the activity of V149L ΔGar1-RNP remains undetectable (Figure 3C and E, Supplementary Table S3). Substitution of V149 with isoleucine, which has a side chain identical in size to that of leucine but similar in shape to that of valine, maintains the reduced catalytic capability of ΔGar1-RNP (Figure 3D, Supplementary Table S3). These results indicate that the catalytic activity is sensitive to the size and shape of the side chain of residue 149. To interrogate the role of chemical property of the residue, we further substituted V149 with three amino acids (V149T, V149D and V149F). Threonine has a side chain with a similar size and shape to valine and V149T ΔGar1-RNP does show a detectable catalytic activity (Figure 3F, Supplementary Table S3). Aspartic acid and phenylalanine have obviously bigger side chains than valine, and the ΔGar1-RNPs assembled by Cbf5 mutants V149D and V149F also completely lost their enzymatic activities (Figure 3G and H, Supplementary Table S3).

**Figure 4.**
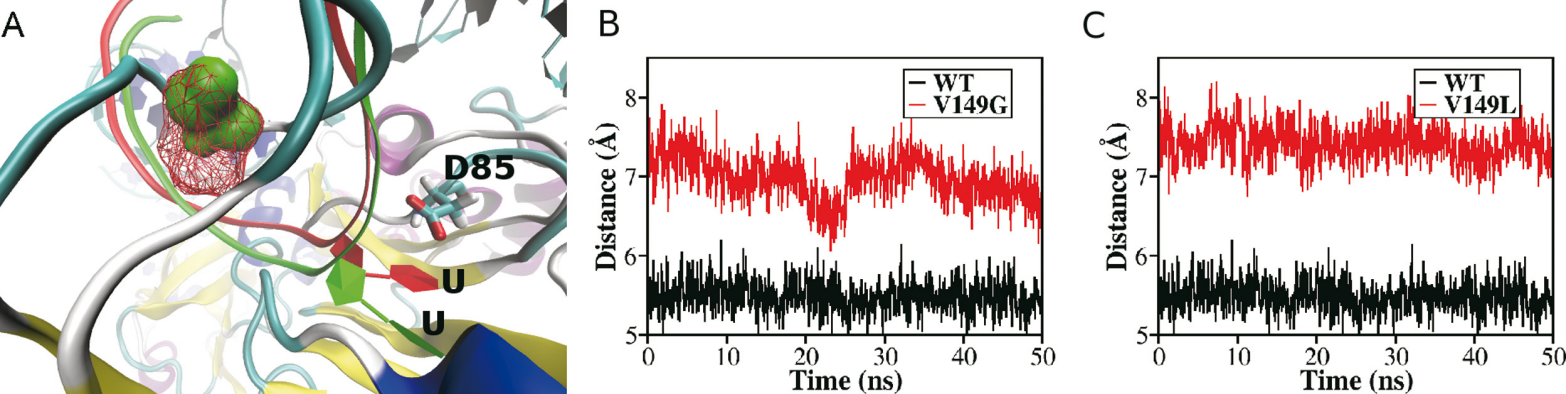
Structural changes induced by mutations of V149. (**A**) The structure alignment of WT-RNP and V149G-RNP. The side chain of V149 (WT-RNP) is shown in red (meshed surface) and the side chain of G149 is shown in green (solid surface). (**B**) The distance between the mass center of target U and the C_γ_ atom of D85: WT-RNP (black) and V149G-RNP (red). (**C**) The distance between the mass center of target U and the C_γ_ atom of D85: WT-RNP (black) and V149L-RNP (red).

These results demonstrated that the steric effect manifested in the size and shape of residue 149 but not the chemical details plays the most important role in determining the catalytic activity of ΔGar1-RNP. To further test this hypothesis, we performed MD simulations for the wild type and V149G or V149L mutant. In Figure 4A, the final structures of WT-RNP and V149G-RNP from the simulations are aligned and compared. Due to the smaller side chain of glycine (green, solid surface) than that of valine (red, meshed surface), the substrate RNA in V149G-RNP (green ribbon) takes a position different from that in WT-RNP (red ribbon). Especially, the tip region of the substrate in V149G-RNP moves away from D85. To quantify such a structural change, we calculated the distances between the C_1_’/C_6_ atom of target U and the C_γ_ atom of D85, and they are shown in Supplementary Figure S2A and B. In both cases, the distances were enlarged by V149G mutation. However, our calculation does not have the ability to tell which carbon that D85 prefers to attack (13–16). Therefore, we took the distance between the mass center of U and the C_γ_ atom of D85 as a suitable indicator for the structural difference. As expected, the V149G mutation resulted in a larger separation between the mass center of target U and C_γ_ atom of D85 than that in WT-RNP, due to the smaller side chain of glycine (Figure 4B). This structural change suggests that a too small side chain of the residue 149 fails to position target U properly in the active site. When we changed the residue V149 to leucine (which has a larger side chain than valine), the distance between the mass center of target U and C_γ_ atom of D85 also became larger than that of WT-RNP (Figure 4C). U is pushed deeper into the pocket and passes D85 (Figure 4A).

### The active site hosts reactant and product differently

**Figure 5.**
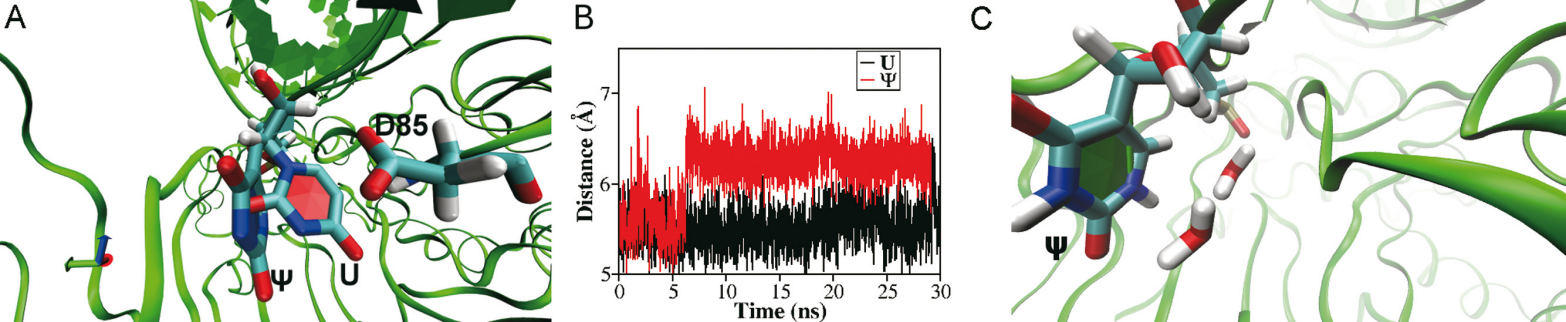
The different conformations of reactant and product in the active site. (**A**) The structure alignment of Sub-U and Sub-Ψ and their orientation relative to D85. (**B**) The distances between C_γ_ atoms of D85 and the mass center of Sub-U (black) and Sub-Ψ (red). (**C**) Two water molecules penetrate into the active site and form hydrogen bonds with Ψ and the sugar phosphate in the preceding residue.

**Figure 6.**
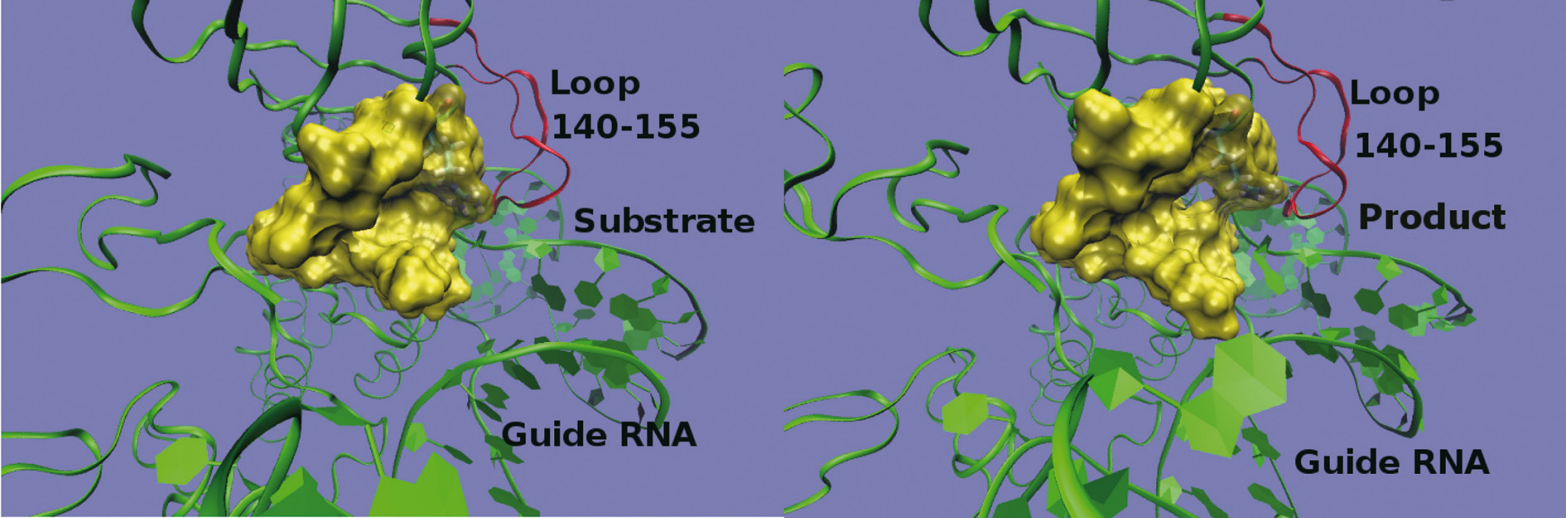
Formation of a hole at the entrance of the active site in the product complex. After U (left) is modified to Ψ (right), the conformation of the active site is changed accordingly which is induced by the rotation of the sugar ring of Ψ. A hole is formed at the entrance of the active site, allowing the water molecules to enter. The residues near the active site are presented with yellow surfaces and the loop is showed with red ribbon.

The dissociation of Sub-Ψ from ΔGar1-RNP is faster than that of Sub-U, and the difference is even larger in the presence of Gar1 (Figure 2C). These results demonstrate that RNP can distinguish between the two molecules. We performed MD simulations to search for the structural origin of the difference in the binding of Sub-Ψ and Sub-U to RNP. Some representative structures are shown in Figure 5A. The simulations showed that the unmodified nucleotide but not the modified one is in close contact with the catalytic amino acid D85 (Figure 5B), and U but not Ψ fits well in the active site. In an earlier study, Neumann *et al*. compared the conformations of uridine and pseudouridine using the proton-proton Overhauser effect and found that pseudouridine takes mainly the syn and uridine the anti conformations (46). Although in our MD simulations the initial structures for both Sub-U and Sub-Ψ were taken to be in the anti-form (the torsion angle $\chi _{\rm 1}$ defined by atom U: O_4_’-C_1_’-N_1_-C_2_ or Ψ: O_4_’-C_1_’-C_5_-C_4_ is ∼130°), the sugar ring of Ψ quickly rotated counterclockwisely around the glycosyl bond by 73°, changing Ψ into the syn-conformation (Supplementary Figure S3). The rotation of the sugar ring subsequently changes the structure of the active site: the side chain of R184 approaches the tip of the loop, allowing two water molecules to enter (Figure 6). One water molecule forms a hydrogen bond with the hydrogen at the N_1_ position of Ψ, and the other forms a hydrogen bond with the sugar phosphate of the polynucleotide chain (Figure 5C). Such a hydrogen-bond-mediated water bridge restricts the conformation of the modified nucleotide and the mobility of the C–C bond, resulting in a more rigid conformation of Ψ (3,5,47). The conformational change induces Sub-Ψ to move out of the binding pocket.

MD simulation trajectories provide additional insight on how the interaction of thumb loop with Gar1 further promotes product release (Figure 2C). In Supplementary Figure S4, we show the root mean square of fluctuation (RMSF) for the backbone atoms of all 16 residues in the thumb loop of the WT-RNP/Sub-Ψ and the ΔGar1-RNP/Sub-Ψ complexes. The RMSF values are larger for the ΔGar1-RNP loop than for the WT-RNP loop, suggesting a higher flexibility of the former. The largest difference of RMSF was observed for residues 140–145, which are all close to Gar1. These findings show that Gar1 interacts with the thumb loop through the root part of the loop (residues 140–145), consistent with the crystal structure study (34). MM/PBSA (45) method was applied to estimate the binding free energy differences of Sub-U and Sub-Ψ for WT-RNP and ΔGar1-RNP (Supplementary Table S4). The results showed that Sub-U binds more strongly with RNP than Sub-Ψ does, no matter whether Gar1 exists or not. Furthermore, the difference of binding free energies between Sub-U and Sub-Ψ when Gar1 is present is notably larger than that when Gar1 is absent. These results, although qualitative, suggested that Gar1 amplifies the difference in dissociation of Sub-U and Sub-Ψ from RNP through modulating the loop conformation triggered by the modification from U to Ψ.

### Gar1 modulates the interaction between substrate RNA and thumb loop

The thumb loop adopts two distinct conformations that depend on the presence of substrate RNA and Gar1 (34,40). In the closed state, the thumb loop binds and stabilizes the fully loaded U substrate, while in the open state the thumb loop moves toward Gar1, and away from the substrate-docking position (34,39,40). MD simulations were performed on both substrate-bound WT-RNP and ΔGar1-RNP. Although due to the limitation of the simulation time, the release of the product was not directly observed, the simulations did allow us to examine the protein/RNA conformational properties under different conditions and provided hints to the enzymatic mechanism. To characterize the opening of the thumb loop which is expected to play an important role in catalytic reaction and product release, the distance between the atom C_α_ of the residue R146 (the tip of the loop) and the atom C_3_’ of the residue Cytosine 602 (RC602) of the guide-RNA was calculated and is shown in Supplementary Figure S5. This figure shows that the distance in ΔGar1-RNP is on average 2 Å shorter than that in WT-RNP. In addition, Supplementary Figure S6 shows that the distance between the mass center of target U and the C_γ_ atom of D85 decreases in the presence of Gar1 compared to that without Gar1, so that D85 is at a proper distance to attack the uridine. Such a distance modulation relies on Gar1's ability of slightly pulling away the thumb loop from substrate RNA. This results is consistent with the experimental findings and suggests that the loop is pulled away from the substrate or product by Gar1, facilitating the modification process and the release of the product. In fact, fluorescence assay using 2-aminopurine (2-AP) has also shown that Gar1 could prohibit the substrate RNA from docking too deep into the active site (38). When Sub-U binds to WT-RNP, the tip of the thumb loop interacts with the substrate RNA, with the root of the thumb loop moved away from Gar1. Under this situation, the attractive interaction at the interface of Gar1 and thumb loop is weak (39). Gar1 has only a minor influence on the Sub-U dissociation from RNP. Due to the interaction between Sub-U and the tip of the thumb loop, Sub-U dissociates slowly (Figure 2C). Upon the U to Ψ isomerization, the interaction between the tip of thumb loop and Sub-Ψ weakens as a result of the structural changes discussed earlier. At the same time, the thumb loop approaches and interacts more favorably with Gar1. To mimic the opening of the thumb loop, we performed MD simulations on WT-RNP with the substrate removed. After the removal of the substrate, the opening of the loop became accelerated, as a result of the elimination of the strong interactions between the substrate and loop/guide-RNA. The distance between R146 and RC602 was calculated and shown in Supplementary Figure S7, which gradually increased as the loop opened up. In addition, the N_ϵ_ atom of R142 was found to form hydrogen bonds with the O_δ_ atom of D380 on Gar1. The distance between R142 and D380 remained small (∼3 Å) in the simulation. In contrast, this distance was large (∼6 Å) and the loop stayed in the closed conformation when the simulation was performed with a loaded Sub-U. These observations support the earlier speculation that after the isomerization of U to Ψ the thumb loop moves toward Gar1, resulting in a stronger interactions between them. After U is converted to Ψ Gar1 pulls the thumb loop further away from Sub-Ψ, to form an almost open conformation and to weaken the interaction between the thumb loop and Sub-Ψ. This mechanism would predict that the thumb loop does not affect strongly the Sub-Ψ dissociation. Accordingly, it would predict a fast dissociation of Sub-Ψ from RNP which is only limited by the interaction between Sub-Ψ and the guide RNA. Indeed, the dissociation rate of Sub-Ψ from the WT-RNP is almost the same with that of DEL7 mutant (37). In the absence of Gar1, the *k*_off_ of loaded Sub-U and Sub-Ψ becomes smaller due to the loss of the pulling effect of Gar1 on the root part of the thumb loop (Figure 2C). Following the above argument one would also expect that Gar1 has a smaller effect on *k*_off_ for Sub-U than for Sub-Ψ, which was exactly what the experiments have shown.

## DISCUSSION

Our results indicate that Gar1 promotes the catalytic reaction entropically. Such an observation can now be rationalized. Stand-alone ΨSs bind the substrate through shape recognition. In RNP, sequence recognition through guide RNA makes a flexible and, at the same time, specific binding machinery possible. To utilize the original architecture of substrate recognition through shape, Nop10 and L7Ae are recruited by Cbf5, to construct the pair of guide-RNA and substrate RNA into a scaffold that matches the shape of the catalytic pocket. However, such a reconstruction of ΔGar1-RNP does not generate the optimized architecture for target U toward D85 in Cbf5. The inclusion of Gar1 allows a fine alignment between target U and D85 through the thumb loop. Our MD simulation results supported such a picture. The distance adjustment exerts a restriction on the configuration of the reactant state (reduction of entropy on the reactive complex, Figure 4A), which leads to the increase of the activation entropy and explains why the reduction on the activation free energy barrier by introducing Gar1 is mainly entropic (Figure 2A). The difference between U and Ψ in the dissociation rates (Figure 2C) supports the previous conclusion that the thumb loop plays an important role in substrate-binding (37). Gar1 can make the thumb loop more rigid and moving synchronously. Gar1 does not affect the dissociation below 22°C, but it enhances the dissociation in WT-RNP at temperatures higher than 22°C through enforcing the loop to move, especially greatly so for the product Sub-Ψ.

The substrate RNA interacts exclusively with the tip region of the thumb loop, which places target U into the active site (26,34). The tip region of the thumb loop anchors the substrate onto the active cleft by the hydrogen bonds formed between the protein backbone atoms and the non-bridging phosphate oxygen of the substrate (34). It was proposed that the modification involves a nucleophilic attack of C_6_ of the uracil ring or C_1_’ of the ribose ring by D85 (13,14,48), or deprotonation of C_2_’ to form a glycal intermediate assisted by D85 as a general base (16). Although our current study could not offer definite information regarding the specific role that D85 plays, it is certain that in all cases, the catalytic D85 and the nucleotide must be placed at a proper position (49). Based on our experimental observations, we realized that the suitable size and shape of V149 is essential for the alignment of target U toward D85, and the mutations perturb the optimal distance between target U and D85 because their side chains are either too small or too large. Our MD results confirm that the steric interaction between substrate RNA and the side chain of V149 plays a subtle but crucial role for the placement of target U to the right position with respect to D85. A rather small deviation from valine (V149A, V149I and V149T) reduces, and further deviation (V149G, V149L, V149D and V149F) abolishes the catalytic activity of the RNP enzyme.

Sub-Ψ and Sub-U have quite similar structures except that Sub-Ψ has an extra hydrogen bond donor, which could interact with the Cbf5 subunit. Such an extra interaction was expected to strengthen the binding of Sub-Ψ with RNP. However, as required by the function of the enzyme, Sub-Ψ binds to RNP more weakly than Sub-U does. Our results demonstrate that the different conformation preferences of U and Ψ induce the conformational changes of the active site. It is most probably that the subsequent conformational change of Cbf5 is the basis for the discrimination of the targets by RNP and causes different dissociation rates of Sub-U and Sub-Ψ. Gar1 makes use of the thump loop to optimize the positioning of substrate U in the active site. It also facilitates the product release, with a minor effect on reactant Sub-U dissociation. Our results suggest that Gar1 amplifies the difference in dissociation of Sub-U and Sub-Ψ from RNP through modulating the loop conformation triggered by the modification from U to Ψ. It is interesting to observe that the hydrogen bonding rearrangement and conformational change of the active site lead to the differentiation between U and Ψ by the enzyme, especially the more favored product release.

The sample RNP in our study came from the thermophile *Pyrococcus furiosus*. It is pity that our technique cannot be applied to the living conditions of *P. furiosus*. However, a speculation based on our data shows that at the living temperatures the differentiation between U and Ψ by RNP would be more substantial and that the catalytic rate would be much faster. For instance, from Figure 2 and by extrapolation, it is estimated that *k*_on,S_, *k*_on,P_, *k*_off,S_, *k*_off,P_, and *k*_cat_ are 4.5 × 10^4^ M^−1^ s^−1^, 4.0 × 10^4^ M^−1^ s^−1^, 0.0013 s^−1^, 0.035 s^−1^, and 0.011 s^−1^ at 30°C, and 5.6 × 10^6^ M^−1^ s^−1^, 1.1 × 10^7^ M^−1^ s^−1^, 0.035 s^−1^, 211 s^−1^, and 4.5 s^−1^ at 60°C, respectively. Although the rates measured at the ambient temperatures are slow, the extrapolated catalytic rates seem much more functionally reasonable at the growth temperature of *Pyrococcus furiosus*, and the relation among these rates matches the desired biological functions better.

## Supplementary Material

SUPPLEMENTARY DATA
