# Supplementary material for: Accurate placement of substrate RNA by Gar1 in H/ACA RNA-guided pseudouridylation
Source: Nucleic Acids Res. 2015 Jul 22;43(15):7207–16. doi: 10.1093/nar/gkv757 (PMC4551948; doi:10.1093/nar/gkv757)
Supplement: SUPPLEMENTARY DATA [file supp_43_15_7207__index.html]

Accurate placement of substrate RNA by Gar1 in H/ACA RNA-guided pseudouridylation — SUPPLEMENTARY DATA 

# Accurate placement of substrate RNA by Gar1 in H/ACA RNA-guided pseudouridylation

## SUPPLEMENTARY DATA

- SUPPLEMENTARY DATA
