# Supplementary material for: Accurate placement of substrate RNA by Gar1 in H/ACA RNA-guided pseudouridylation
Source: Nucleic Acids Res. 2015 Jul 22;43(15):7207–16. doi: 10.1093/nar/gkv757 (PMC4551948; doi:10.1093/nar/gkv757)
Supplement: SUPPLEMENTARY DATA [file supp_gkv757_nar-00983-r-2015-File008.docx]

Supporting Information for

**Accurate Placement of Substrate RNA by Gar1 in H/ACA RNA-guided Pseudouridylation**

# Peng Wang, Lijiang Yang, Yi Qin Gao*, Xin Sheng Zhao*.

## College of Chemistry and Molecular Engineering, Beijing National Laboratory for Molecular Sciences, State Key Laboratory for Structural Chemistry of Unstable and Stable Species, and Biodynamic Optical Imaging Center (BIOPIC), Peking University, Beijing 100871, China

* To whom correspondence should be addressed. Tel: +86 10 62751727; Fax: +86 10 62751708; Email: zhaoxs@pku.edu.cn

Correspondence may also be addressed to Yi Qin Gao, Tel: +86 10 62752431; Fax: +86 10 62751708; Email: gaoyq@pku.edu.cn

The authors wish it to be known that, in their opinion, the first two authors should be regarded as joint First Authors.

**Table S1.** The activation energies of reaction, substrate or product association and dissociation.

| Protein | *E*_a_(kcal mol^-1^)  Reaction | *E*_a_(kcal mol^-1^)  Substrate Association | *E*_a_(kcal mol^-1^)  Product Association | *E*_a_(kcal mol^-1^)  Substrate Dissociation | *E*_a_(kcal mol^-1^)  Product Dissociation |
| --- | --- | --- | --- | --- | --- |
| WT-RNP | 40.4 ± 4.5 | 32.2 ± 5.8 | 37.8 ± 5.8 | 21.9 ± 1.9 | 58.2 ± 4.0 |
| ΔGar1-RNP | 40.7 ± 7.1 | 30.2 ± 4.6 | 33.4 ± 4.1 | 28.8 ± 3.5^a^  0.26 ± 0.16^b^ | 63 ± 10^a^  18.3 ±1.1^b^ |

The errors are from fitting.

^a^ Obtained by fitting the data below 22 °C.

^b^ Obtained by fitting the data above 22 °C .

**Table S2.** The kinetic parameters among different RNP mutants and substrate or product.

| RNP | *k*_on,S_  (10^3^ M^-1^ s^-1^)  22 ^o^C | *k*_off,S_  (10^-3^ s^-1^)  22 ^o^C | *k*_on,P_  (10^3^ M^-1^ s^-1^)  22 ^o^C | *k*_off,P_  (10^-3^ s^-1^)  22 ^o^C | ΔGar1  RNP | *k*_on,S_  (10^3^ M^-1^ s^-1^)  27 ^o^C | *k*_off,S_  (10^-3^ s^-1^)  37 ^o^C | *k*_on,P_  (10^3^ M^-1^ s^-1^)  27 ^o^C | *k*_off,P_  (10^-3^ s^-1^)  37 ^o^C |
| --- | --- | --- | --- | --- | --- | --- | --- | --- | --- |
| WT | 8.8 ± 1.6 | 0.56 ± 0.03 | 5.9 ± 0.6 | 3.51 ± 0.06 | WT | 25.9 ± 2.1 | 0.63 ± 0.01 | 22.3 ± 2.0 | 14.1 ± 1.0 |
| R146G | 4.5 ± 0.2 | 1.39 ± 0.01 | 2.5 ± 0.1 | 1.31 ± 0.02 | R146G | 21.7 ± 0.8 | 1.75 ± 0.04 | 23.2 ± 5.0 | 11.2 ± 1.2 |
| S147G | 5.9 ± 0.8 | 1.41 ± 0.03 | 4.7 ± 0.1 | 1.64 ± 0.06 | S147G | 23.5 ± 2.9 | 1.97 ± 0.05 | 19.7 ± 1.3 | 8.4 ± 0.4 |
| A148G | 6.1 ± 0.2 | 1.68 ± 0.02 | 6.1 ± 0.9 | 1.42 ± 0.05 | A148G | 12.5 ± 0.8 | 1.31 ± 0.02 | 25.2 ± 3.8 | 6.67 ± 0.2 |
| V149G | 4.7 ± 0.8 | 1.33 ± 0.02 | 4.9 ± 0.3 | 1.81 ± 0.04 | V149G | 17.4 ± 1.0 | 1.80 ± 0.05 | 29.3 ± 4.9 | 8.6 ± 0.4 |
| V149A | 4.6 ± 0.5 | 1.62 ± 0.03 | 4.8 ± 0.6 | 1.18 ± 0.05 | V149A | 25.7 ± 3.4 | 1.81 ± 0.05 | 24.9 ± 1.8 | 14.7 ± 5.7 |
| V149L | 3.9 ± 0.2 | 1.61 ± 0.04 | 6.4 ± 1.2 | 1.55 ± 0.05 | V149L | 23.5 ± 2.2 | 2.24 ± 0.09 | 19.9±2.5 | 11.3 ± 2.0 |
| K150G | 4.6 ± 0.1 | 1.67 ± 0.04 | 3.5 ± 0.4 | 1.46 ± 0.06 | K150G | 29.0 ± 2.7 | 2.68 ± 0.06 | 15.4 ± 2.2 | 20.4 ± 3.3 |
| R151G | 5.4 ± 0.7 | 1.62 ± 0.04 | 4.5 ± 0.3 | 1.16 ± 0.03 | R151G | 20.7 ± 1.1 | 2.56 ± 0.12 | 22.2 ± 6.1 | 15.4 ± 3.2 |

The errors of *k*_on_ are from fitting.

The errors of *k*_off_ are from 2-3 independent measurements.

**Table S3**. Modification rates of different ΔGar1-RNP mutants at 37 ^o^C.

| Mutants | WT | R146G | S147G | A148G | V149G | V149A | V149I | V149L | V149T | V149D | V149F | K150G | R151G |
| --- | --- | --- | --- | --- | --- | --- | --- | --- | --- | --- | --- | --- | --- |
| *k*_cat_  (10^-3^ s^-1^) | 3.80 ± 0.65 | 0.95 ± 0.21 | 0.23 ± 0.07 | 0.32 ± 0.08 | tstd | 0.13 ± 0.03 | 0.11 ± 0.03 | tstd | 0.11 ± 0.02 | tstd | tstd | 0.23 ± 0.03 | 1.57 ± 0.37 |

The errors are from 2-3 independent measurements.

tstd: too slow to be detected in 5 h.

**Table S4**. The binding free energies (kcal mol^-1^) of U and Ψ obtained from MM/PBSA calculations.

| Protein | U | Ψ |
| --- | --- | --- |
| WT-RNP | -9.50 | 5.56 |
| ΔGar1-RNP | -4.05 | -0.06 |


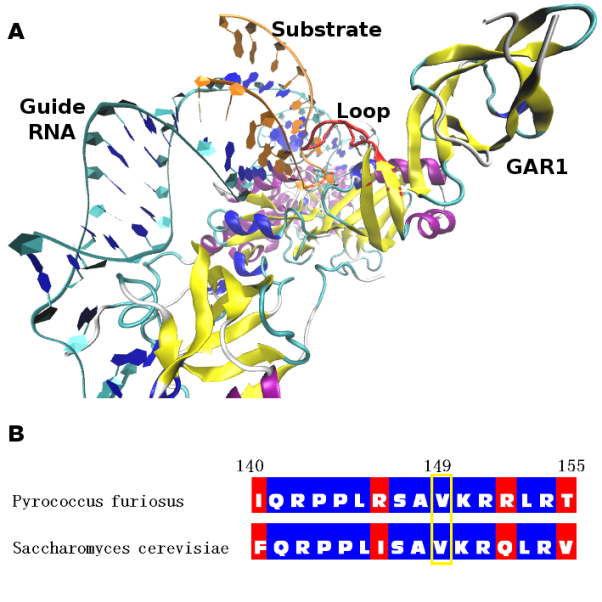


**Figure S1.** The closed conformation of the thumb loop. (A) The thumb loop interacts extensively with the substrate in the closed conformation. (B) The sequence alignment of Cbf5s (loop region: residue 140-155) from archaeal *pyrococcus furiosus* and eukaryotic *saccharomyces cerevisiac*.


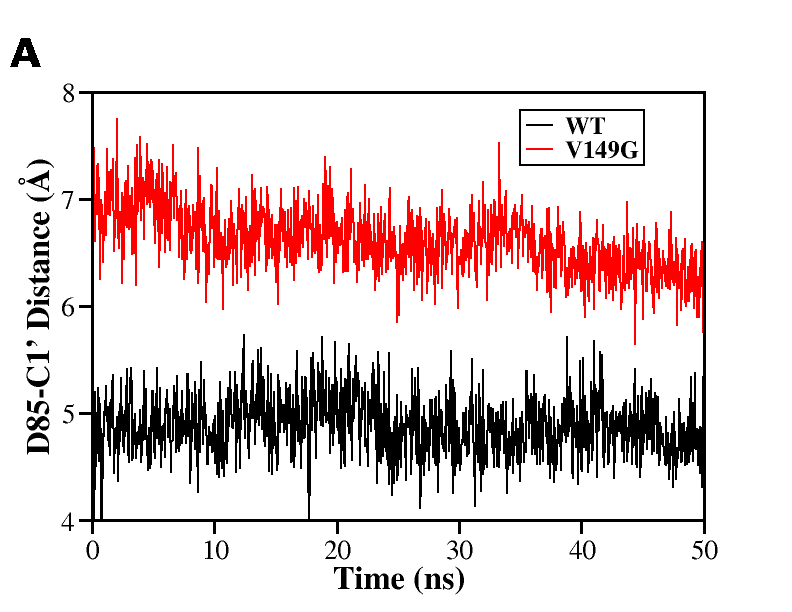


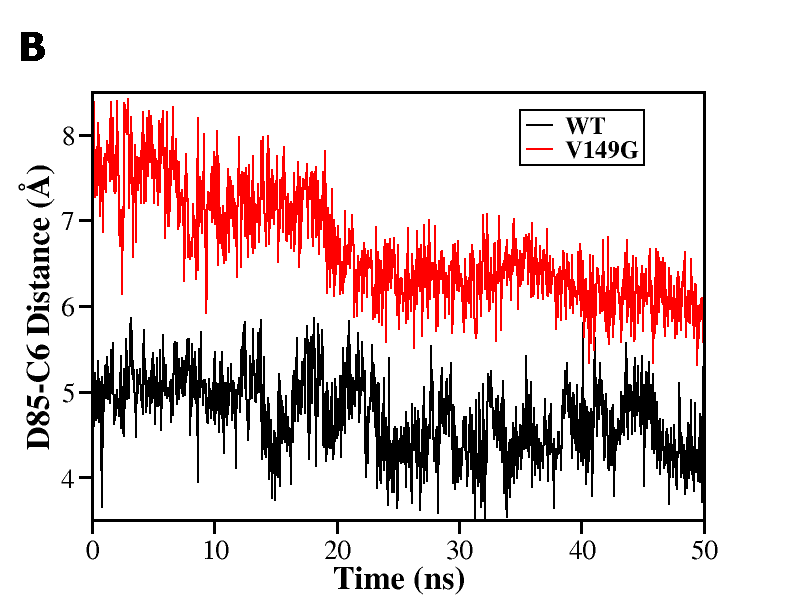


**Figure S2** Structural changes induced by mutations of V149. (A) The distance between the C_1_’ atom of target U and the C_γ_ atom of D85: WT-RNP (black) and V149G-RNP (red). (B) The distance between the C_6_ atom of target U and the C_γ_ atom of D85: WT-RNP (black) and V149G-RNP (red).


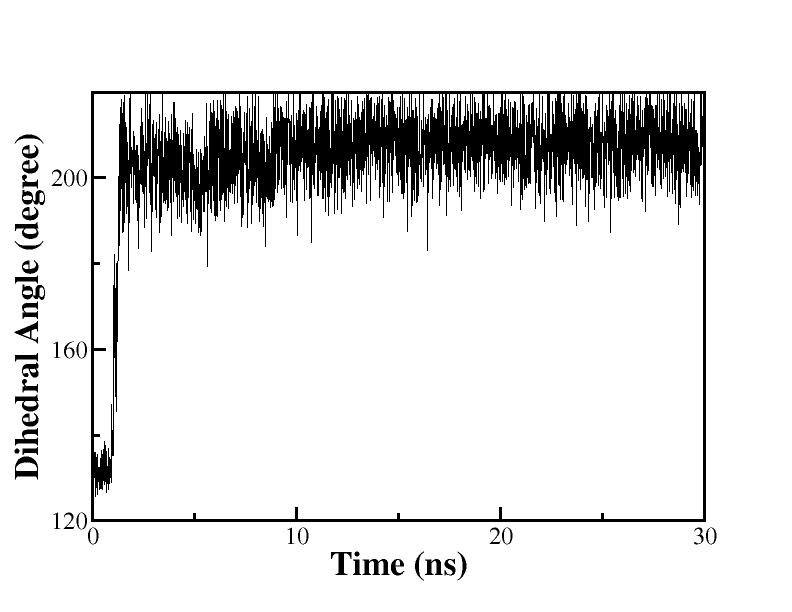


**Figure S3**. The rotated sugar ring of Ψ. Starting from the anti- conformation, the sugar ring quickly rotated 73° counterclockwisely around the glycosyl bond to assume the syn-conformation.


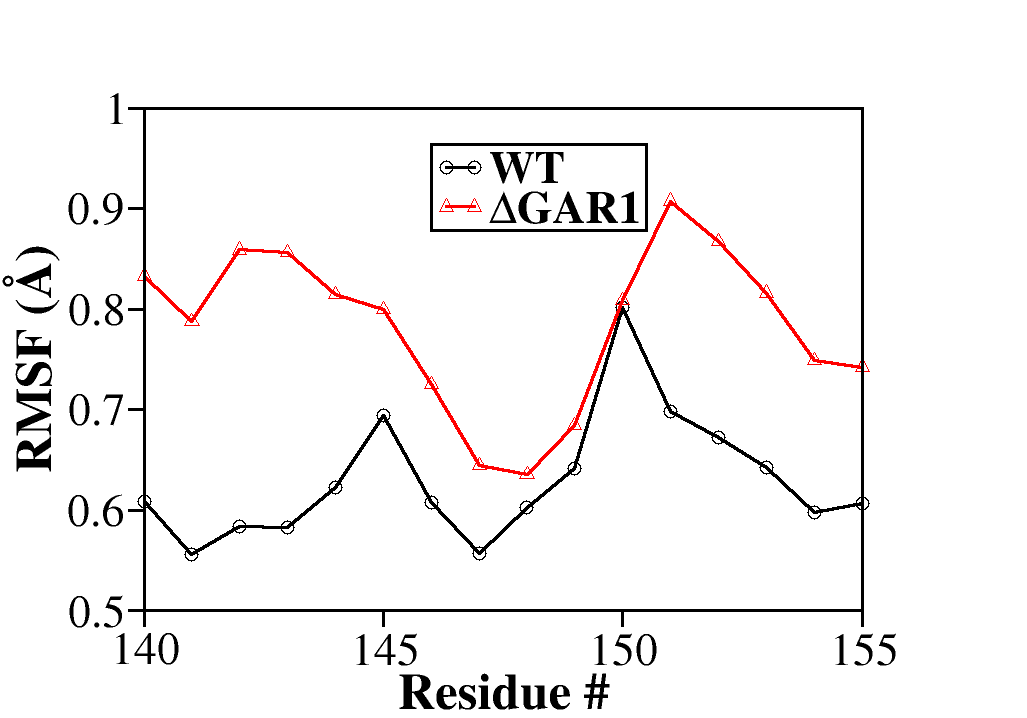


**Figure S4.** The atomic positional fluctuations for the backbone atoms of all 16 residues (140-155) on the thumb loop: WT-RNP/Sub-Ψ (black) and ΔGar1-RNP/Sub-Ψ (red).

**
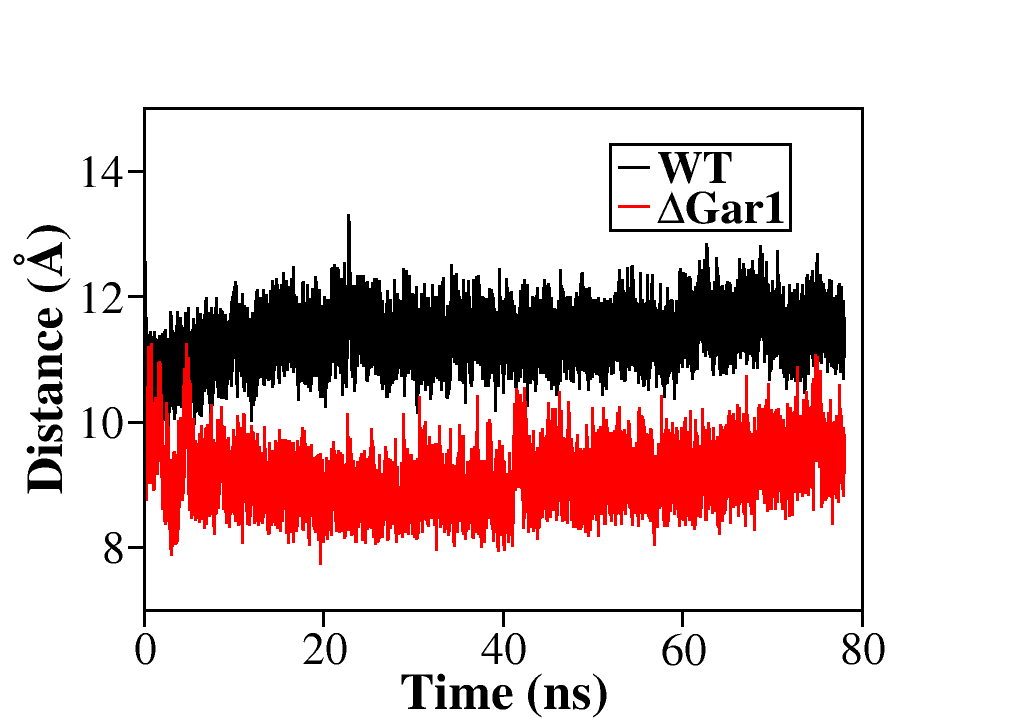
**

**Figure S5.** The distance between C_α_ of R146 (the tip of the thumb loop) and C_3_’ of residue Cytosine 602 (RC602) on guide RNA measured in MD simulations. The distances obtained from WT-RNP/Sub-Ψ are represented by the black lines and the ones obtained from ΔGar1-RNP/Sub-Ψ are represented by the red lines.

**
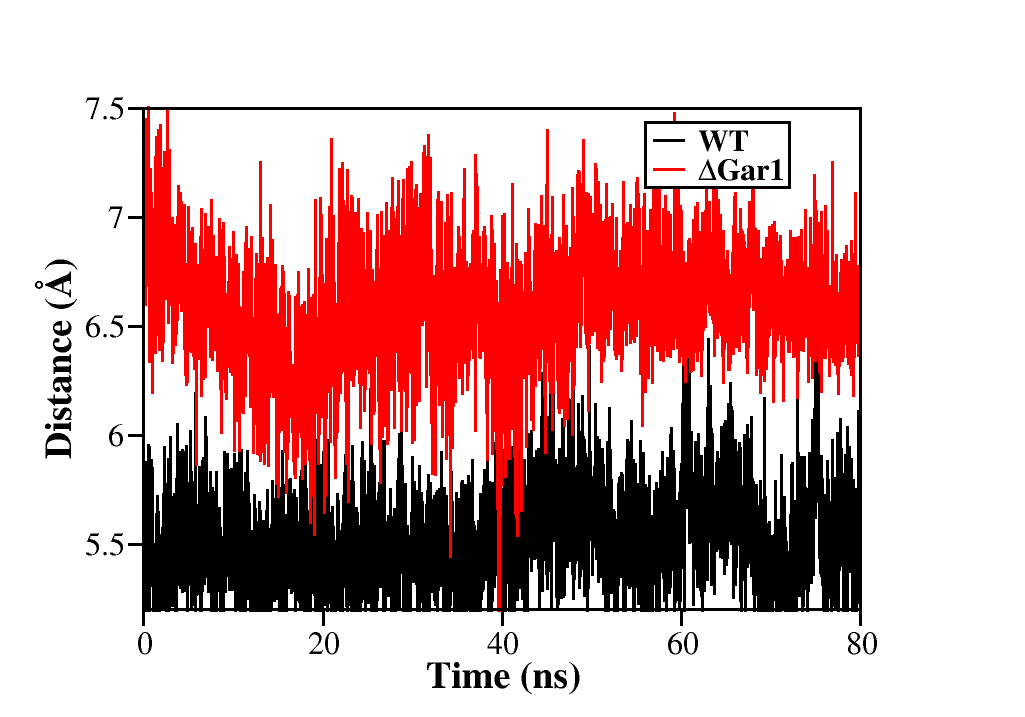
**

**Figure S6.** The distance between the mass center of target U and C_γ_ atom of D85: WT-RNP (black) and ΔGar1-RNP (red).

**
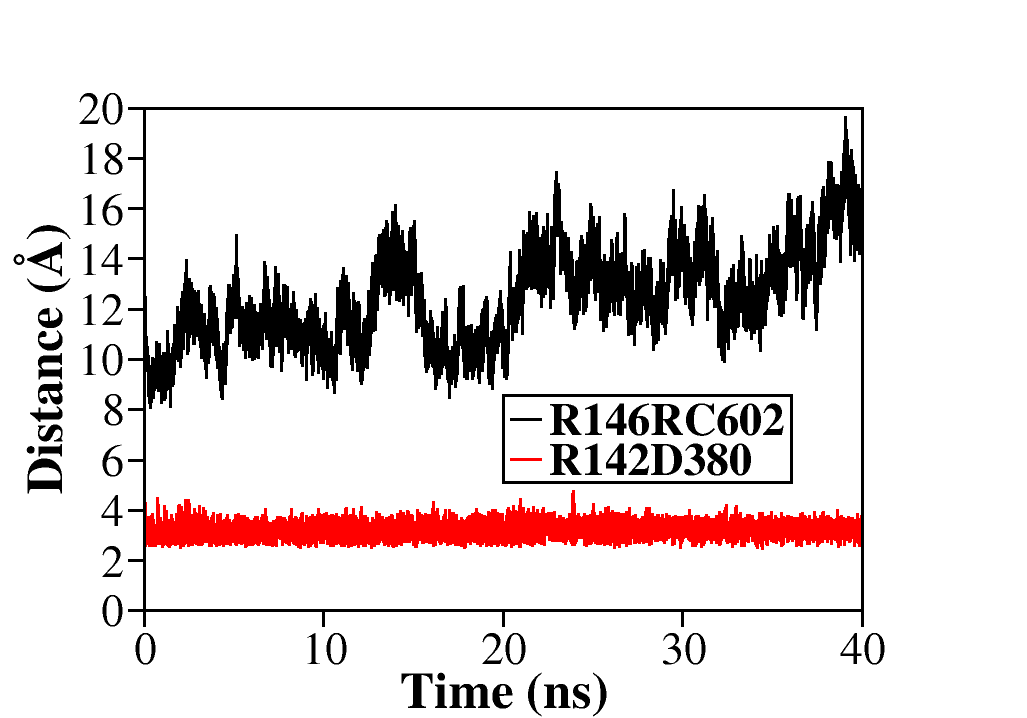
**

**Figure S7.** The distances between C_α_ of R146 (the tip of the thumb loop) and C_3_’ of residue Cytosine 602 (RC602) on guide RNA (black) and between N_ε_ atom of R142 on the thumb loop and O_δ_ atoms of D380 on Gar1 (red). Hydrogen bond can form between N_ε_ and O_δ_. The MD simulations were conducted on WT-RNP with the substrate removed to accelerate the opening of the thumb loop.
